# Influence of Disease-Related Stigma on Patients’ Decisions to Upload Medical Reports to the German Electronic Health Record: Randomized Controlled Trial

**DOI:** 10.2196/52625

**Published:** 2024-04-10

**Authors:** Niklas von Kalckreuth, Markus A Feufel

**Affiliations:** 1 Division of Ergonomics Department of Psychology and Ergonomics Technische Universität Berlin Berlin Germany

**Keywords:** electronic health record, EHR, technology acceptance, upload behavior, health-related stigma, intention to use, intention-behavior gap, medical reports, stigma, Germany, patient decision, digital transformation, implementation, risk, decision, risk perception, social stigma, safety

## Abstract

**Background:**

The rollout of the electronic health record (EHR) represents a central component of the digital transformation of the German health care system. Although the EHR promises more effective, safer, and faster treatment of patients from a systems perspective, the successful implementation of the EHR largely depends on the patient. In a recent survey, 3 out of 4 Germans stated that they intend to use the EHR, whereas other studies show that the intention to use a technology is not a reliable and sufficient predictor of actual use.

**Objective:**

Controlling for patients’ intention to use the EHR, we investigated whether disease-specific risk perceptions related to the time course of the disease and disease-related stigma explain the additional variance in patients’ decisions to upload medical reports to the EHR.

**Methods:**

In an online user study, 241 German participants were asked to interact with a randomly assigned medical report that varied systematically in terms of disease-related stigma (high vs low) and disease time course (acute vs chronic) and to decide whether to upload it to the EHR.

**Results:**

Disease-related stigma (odds ratio 0.154, *P*<.001) offset the generally positive relationship between intention to use and the upload decision (odds ratio 2.628, *P*<.001), whereas the disease time course showed no effect.

**Conclusions:**

Even if patients generally intend to use the EHR, risk perceptions such as those related to diseases associated with social stigma may deter people from uploading related medical reports to the EHR. To ensure the reliable use of this key technology in a digitalized health care system, transparent and easy-to-comprehend information about the safety standards of the EHR are warranted across the board, even for populations that are generally in favor of using the EHR.

## Introduction

### Background

The digital transformation of health care promises safety and efficiency gains by connecting all players in a health care system [1-3]. One key technology to connect health professionals, insurance providers, and patients is the electronic health record (EHR), which will be implemented nationwide and mandatory for all patients in Germany starting on January 1, 2025. In the EHR, patients’ medical data (eg, findings, diagnoses, therapies, vaccinations, discharge reports, emergency data, and medication plans [4,5]) can be digitally documented, exchanged, and viewed [4,6]. Better coordination of health data can ultimately save costs in the health care system [7-9].

In Germany, the Patient Data Protection Act [10] mandates that it is ultimately the patient who controls the type of data that are stored and can be viewed in the EHR. Although a recent survey found that 3 out of 4 Germans state that they intend to use the EHR [11], its success ultimately depends on whether and under what circumstances it is actually *used* to store and share health data. As described below based on the available literature, intention to use is *not* a sufficient and reliable predictor of EHR use. Therefore, in this study, we sought to investigate to what extent intention to use predicts actual use and what additional factors may need to be taken into account to more reliably predict EHR use.

### Related Work

The technology acceptance model (TAM) and its extensions such as the unified theory of acceptance and use of technology (UTAUT) assume a positive relationship between intention to use a technology (technology acceptance) and actual use [12-14]. In fact, empirical studies on social networks and online banking show that the greater the intention to use, the more likely the technology will actually be used. However, the same studies also show a statistical discrepancy between intention and behavior, as evidenced by the different variance (*R*²) accounted for by the two constructs [15-17]. Questionnaire studies on this so-called “intention-behavior gap” suggest that intention is not a reliable predictor of behavior and consequently that other influencing factors must exist [18,19]. For instance, in the context of social media and electronic commerce, users often have massive privacy concerns to disclose their data and their intentions to use are generally low. Nonetheless, users tend to disclose their data if the benefits they expect from using the applications are sufficiently high [20]; this phenomenon is called the “privacy paradox” and has been confirmed repeatedly [15,20,21]. However, questionnaire studies on digital health technologies show no such paradox and more nuanced patterns. For health technologies, privacy concerns thus far either had no influence [22-24] or have been shown to have a systematic negative impact on intentions and actual technology use [25,26]. In summary, based on the available research, it is unclear to what extent intention to use predicts the actual use of digital health technologies such as the EHR. Theories of technology acceptance infer a direct, positive influence, whereas the results of various questionnaire studies suggest that other factors must play a role given the intention-behavior gap. Although the influence of a few technology-related factors (eg, controllability of data) on the intention to use an EHR have been investigated, a thorough investigation of disease-related factors has not yet been performed.

Methodologically, usage behavior has mostly been investigated using self-report questions about the frequency of use [15,16,27-29], which is associated with several limitations. First, frequency of use is only meaningful if the system is already established and widely used. In the case of new systems such as the EHR in Germany, frequency of use cannot be surveyed. Second, the actual context of use can be difficult to simulate in questionnaire studies, making it difficult to distinguish between intention and behavior [30]. Since the models of technology acceptance described above (ie, TAM and UTAUT) have been evaluated using questionnaires, they may not provide reliable insights into usage behavior in the context of the EHR.

Therefore, to investigate usage behavior regarding the EHR in Germany, we selected a different approach for this study. In terms of uploading behavior, we first identified two possible use cases: (1) users who are living with different acute as well as chronic diseases (“patients with multimorbidity” use case), enabling a direct comparison between different medical findings in terms of risks and benefits of uploading to the EHR; and (2) users who are healthy or have little to no preexisting conditions before they develop a chronic or acute disease (“patients with first contact” use case). To investigate these use cases, we developed and used an interactive prototype of the EHR (ie, a click dummy) to investigate factors influencing the EHR users’ decision to upload medical reports. Compared to questionnaire studies, this approach has the advantage that the interaction with the click dummy is closer to a real interaction with the EHR, thereby increasing the ecological validity of behavioral measures [30]. To investigate the first use case, we used a mixed methods design where the experimental intervention was based on an interview study with potential EHR users [31]. The interview study showed that the time course of a disease (chronic vs acute) and disease-related stigma influence people’s decisions to upload a medical report to the EHR. The following experiment showed that respondents were more likely to upload a medical report of a chronic disease to the EHR than to upload a report of an acute condition. In contrast, respondents were less likely to upload a report of a disease with high stigma. When a disease with high stigma had a chronic time course, reports were still uploaded. We here report the results of the second use case in which participants interacted with one medical report only.

## Methods

### Ethical Considerations

This study was approved by the Ethics Committee of the Department of Psychology and Ergonomics (Institut für Psychologie und Arbeitswissenschaft) at Technische Universität (TU) Berlin (tracking number: AWB_KAL_1_230311). Participants volunteered to participate in the survey and informed consent was required. On the first page of the survey, participants were told about the investigator, the study purpose, what data were to be collected during the study, and where and for how long they would be stored. Participants were informed about the duration of the survey (approximately 8 minutes) as well as the compensation for participation. Participants were compensated with €1.60 (US $1.75) for their time and thus according to minimum wage. The participants also had the possibility to download a PDF of the participant information on the first page.

Participants’ personal data and responses were kept entirely anonymous and password-protected in the department’s data vault. An anonymized data set from the study was made available to other researchers for further analysis with open access. The documentation and availability of the research data collected during the study were managed using the TU repository “DepositOnce,” adhering to the regulations for ensuring good scientific practice at TU Berlin, the guidelines of the “DepositOnce” internal research data repository, and data protection regulations. Compliance with these repository guidelines ensures the indexing and findability of the research data by third parties.

### Participants

The study was conducted from May 9 to June 10, 2023. Based on an a priori power analysis for a logistic regression with three predictors as well as a false-positive rate α of .05 and a power of 1–β=0.80, we aimed for a sample size of 186 participants. Individuals 18 years and older residing in Germany were eligible to participate in the study. Another prerequisite was that participants had no previous personal experience (own illness) with the diseases mentioned in the medical reports, as affected people deal with disease-related stigma differently than people who are not affected by the disease [32]. Sampling was conducted through Prolific [33], a click worker platform characterized by high data quality [34]. A total of 275 individuals participated in the study. The mean participation time was 9 minutes, 28 seconds (SD 3 minutes, 47 seconds) and the median was 8 minutes, 36 seconds.

### Design

We used a 2×2 between-subject study design with the independent variables stigma (high vs low) and time course of illness (chronic vs acute). Stigma was operationalized as the risk that the medical findings could negatively affect the private, professional, or social life of the affected person. For this purpose, the medical reports related to personal lifestyle, as reflected in tests for sexually transmitted diseases [31,32]. The time course is a classification of diseases in terms of their duration. These can be either acute (diseases of short duration that come on quickly) or chronic (diseases that develop slowly or last for a longer time). The dependent variable was the decision to upload the medical report (ie, whether participants were willing to upload the medical findings to the EHR). Furthermore, the intention to use the EHR was included as a covariate.

### Materials

The stimuli used in the study were realistic but specially created for the purpose of the study. The medical reports were provided by various hospitals and a medical association. To make the reports appear as realistic as possible, they were edited on the official document heads of these institutions (see Multimedia Appendix 1). In selecting the diseases, both the related stigma and time course were systematically varied. To reflect different disease-related stigma, which covered different risks for professional and social life [35-38], diseases were divided according to their low and high stigmatization potential. To reflect different time courses, diseases were divided according to an acute and chronic time course. Furthermore, diseases were selected to occur regardless of age so that they would be perceived as realistic diseases by an age-diverse sample. Table 1 shows the diseases used as stimuli, categorized by level of stigma potential and time course.

**Table 1:** Diseases used in the stimuli, categorized by level of stigma potential and time course.

Stigma potential	Acute disease	Chronic disease
Low	Fractured wrist	Type 1 diabetes
High	STD^a^ (gonorrhea)	Depression

^a^STD: sexually transmitted disease.

The interface software FIGMA was used to create the click dummy, which was modeled after the mobile EHR app of a German health insurance company (BARMER)—the eCare app—to support a realistic interaction with an EHR. Specifically, the click dummy allowed participants to upload findings, grant or revoke permissions to view medical reports, and create medication plans. Only the “Upload report” function was used in this study.

We used LimeSurvey (version 3.28.3+220315) to create and conduct a 5-page online survey (see Multimedia Appendix 2). The EHR click dummy and the medical reports were incorporated into the survey using iFrame. LimeSurvey software was used to ensure that all questions had to be answered to complete the study and receive the compensation. As in the previous study investigating the first use case [31], in this study, we tested the effect of the independent variables by querying the perceived privacy risk and perceived benefit of uploading findings to the EHR as manipulation checks. Based on the results of this previous study [31], we assumed that high stigma would result in a high perceived privacy risk and a chronic time course would result in a high perceived benefit of uploading the medical report. Perceived privacy risk, perceived benefit, and intention to use were measured using a 7-point Likert scale ranging from 1 (“strongly disagree”) to 7 (“strongly agree”). The decision to upload the finding to the EHR was measured using a dichotomous item (yes/no).

### Procedure

**Figure 1:**
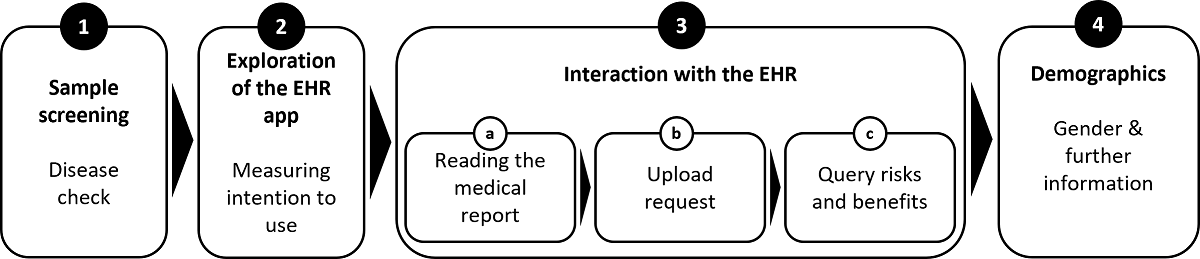
Overview of the study procedure. EHR: electronic health record.

The study procedure is shown schematically in Figure 1. Before the start of the experiment, participants gave their informed consent. This was followed by screening questions related to disease experience (step 1). Participants who had experience with the diseases in the medical reports were excluded from the study. Subsequently, participants were given 1 minute to interact with the EHR click dummy and were then required to answer questions regarding their intention to use the EHR (step 2). Participants were then asked to interact with the medical report (step 3). In this process, each person was first randomly assigned to one of the four diseases shown in Table 1 and asked to read an easy-to-understand description of the disease of approximately 2-3 sentences (see Multimedia Appendix 3) (step 3a). Participants then decided whether they wanted to upload the report to their EHR (step 3b). Afterward, participants were asked to rate the perceived privacy risks and benefits of uploading the report (step 3c). The survey was completed with the collection of demographic characteristics (age, gender, education level, and experience with EHRs). In this step (step 4), the participants also had the opportunity to declare their responses invalid, while still receiving compensation, in case they did not pay sufficient attention to the instructions provided (eg, due to choosing random answers, inattentively reading questions, or rushing through the survey).

### Analysis

We cleaned and analyzed the data using RStudio (version 1.3.1093). Due to lack of variance inhomogeneity or a normal distribution, the analyses regarding perceived privacy risks and benefits were performed using the nonparametric Mann-Whitney *U* test. As mentioned above, we hypothesized that high stigma would result in a high perceived privacy risk and a chronic time course would result in a high perceived benefit of uploading the medical report. The influence of the independent variables (disease-specific stigma and time course) and the covariate “intention to use” on the upload decision were tested using multiple logistic regression with dummy coding. We hypothesized that usage behavior is negatively influenced by disease-specific stigma and positively influenced by time course and intention. To control for demographic and interindividual influences, we used multiple logistic regression with standardized coefficients for better comparability. In doing so, we followed the recommendations for testing control variables [39] and tested the variables that have been shown to be causally related to privacy behavior along with the independent variables. The control variables were age, education level, and experience with the technical system, in this case the EHR [40,41].

## Results

### Sample Characteristics

**Figure 2:**
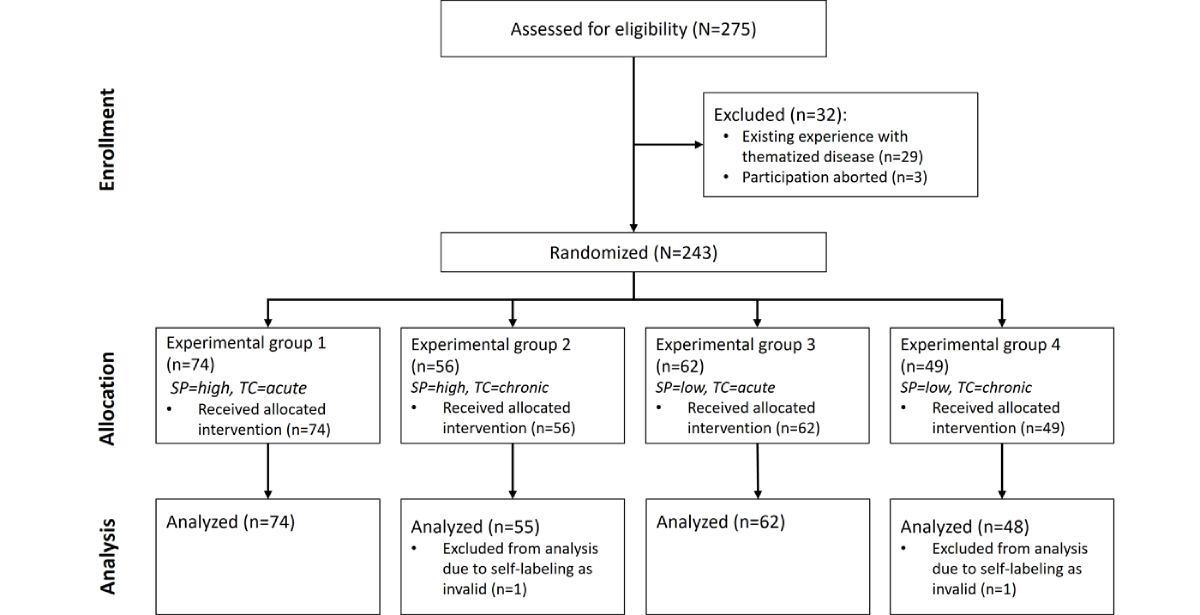
CONSORT (Consolidated Standards of Reporting Trials) flow chart. SP: stigma potential; TC: time course.

A total of 275 observations were collected. Of those, 34 records were excluded, 29 because of participants’ previous medical histories, 3 because of incomplete questionnaires, and 2 because they were marked as invalid by participants. Figure 2 shows the flow of participants in the study based on the CONSORT (Consolidated Standards of Reporting Trials) statement [42].

Thus, a sample of 241 observations (146 male, 92 female, 1 diverse, 2 no information provided) was used for further analysis. Table 2 summarizes the demographic characteristics of the sample.

**Table 2:** Demographic data of the sample (N=241).

Demographic characteristic		Value
Age (years), mean (SD)		31.31 (9.76)
**Gender, n (%)**		
	Female	92 (38.2)
	Male	146 (60.6)
	Other	3 (1.2)
**Education, n (%)**		
	No degree	9 (3.7)
	School leaving certificate	3 (1.2)
	Secondary school certificate	18 (7.5)
	General qualification for university entrance	66 (27.4)
	Vocational training	33 (13.7)
	University degree (bachelor’s or master’s degree)	112 (46.5)
**Experience with the German EHR^a^, n (%)**		
	EHR is unknown	61 (25.3)
	EHR is known but not used	164 (68)
	Occasional use	14 (5.8)
	Regular use	2 (0.8)

^a^EHR: electronic health record.

### Risk and Benefit Perception

**Figure 3:**
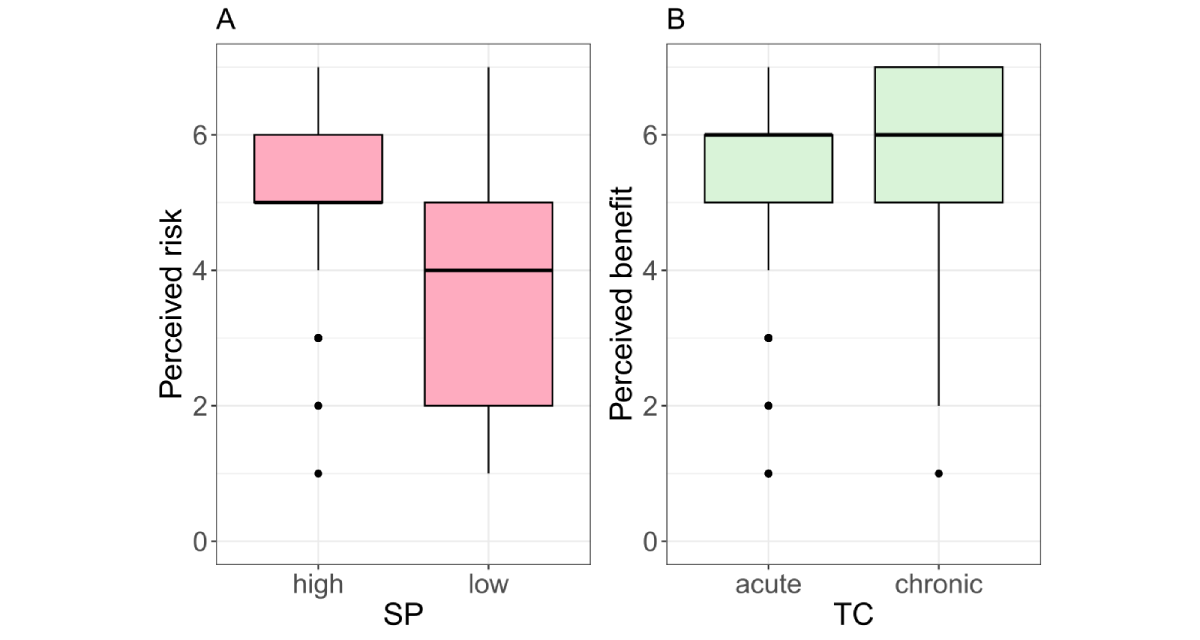
(A) Perceived risk in relation to stigma potential (SP) and (B) perceived benefit in relation to the disease time course (TC). The horizontal line in the box represents the median.

We first checked whether stigma potential had an effect on privacy risk perception and whether time course had an effect on the benefit perception of uploading (see Figure 3). Mann-Whitney *U* tests showed a significant effect of stigma potential on privacy risk perception (*W*=10,777; *P*<.001), where high stigma was associated with high risk. The effect of the disease time course on benefit perception was not significant (*W*=6379; *P*=.14), with a mean benefit perception of 5.34 (SD 1.39) for acute diseases and of 5.54 (SD 1.43) for chronic diseases. Consequently, in contrast to our study on the first use case with several medical reports [31], there was no relationship found between time course and perceived benefits when there is only one report to upload.

### Controls

To investigate the potential association between the decision to upload the medical report and the independent variables disease-specific stigma and time course, we first performed a logistic regression (Hosmer-Lemeshow *R*^2^=0.319, Nagelkerke *R*^2^=0.590, Cox-Snell *R*^2^=0.537; *χ*^2^_15_=86.973; *P*<.001) to control for the covariate intention to use and the demographic variables age, sex, education level, and experience with the EHR. The covariate intention to use (odds ratio [OR] 2.497, 95% CI 1.831-3.456; *z*=5.455; *P*<.001) showed an association with the decision to upload, whereas none of the control variables had an effect. These variables were consequently removed from the model for further analyses.

### Uploading Behavior

To examine the association between the decision to upload and the independent variables stigma potential and time course, we performed a logistic regression controlling for the covariate intention to use (Hosmer-Lemeshow *R*^2^=0.289, Nagelkerke *R*^2^=0.551, Cox-Snell *R*^2^=0.501; *χ*^2^_3_=78.748; *P*<.001). Intention to use was positively associated with uploading behavior; specifically, as intention to use increased, it was more than twice as likely that the report was uploaded to the EHR. In addition, there was a negative association between stigma and the decision to upload; specifically, when stigma was high, it was six times less likely that the report was uploaded than when stigma was low. Time course of the disease was not associated with the decision to upload a report. The summary of the results of the logistic regression are shown in Table 3.

**Table 3:** Results of the logistic regression.

Variable	*z* value	*P* value	Odds ratio (95% CI )
Intention to use	6.210	<.001	2.682 (1.971-3.639)
Stigma potential	4.463	<.001	0.154 (0.064-0.336)
Time course	0.244	.81	1.093 (0.537-2.254)

**Figure 4:**
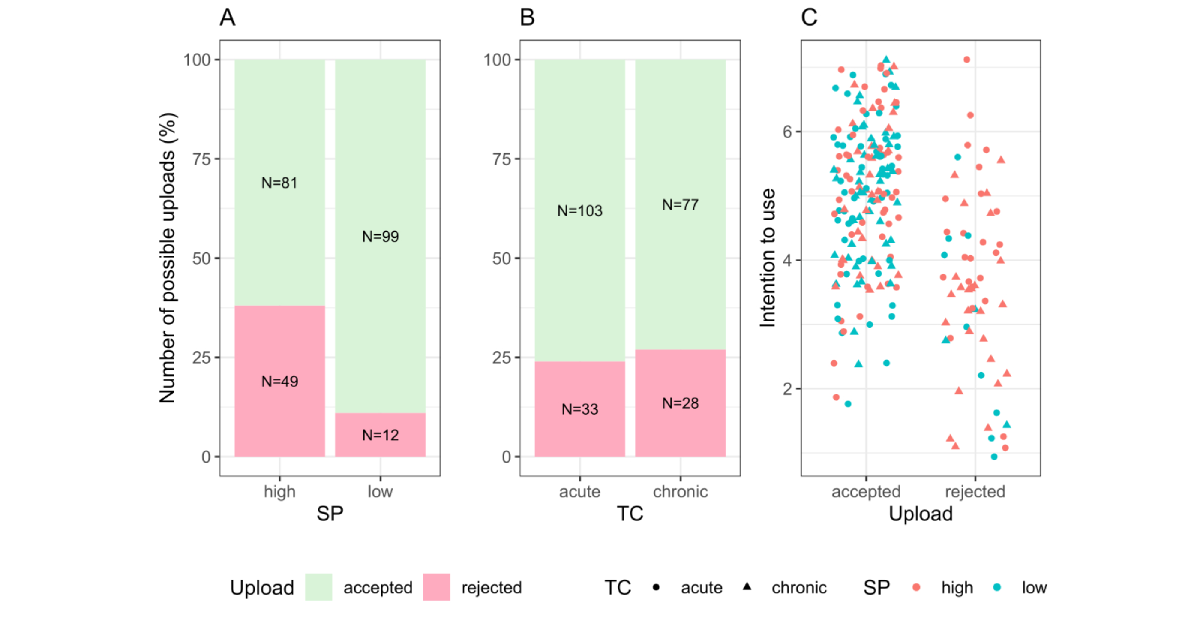
Number of uploads to the electronic health record with respect to (A) stigma potential (SP) and (B) time course (TC), and (C) the influence of intention to use on the decision to upload as a function of SP and TC.

The number of uploads is shown in Figure 4 in relation to the independent variables stigma potential (Figure 4A) and time course (Figure 4B). In addition, we show the relationship between intention to use and the decision to upload a report as a function of the independent variables in Figure 4C.

## Discussion

### Principal Findings

Our results show that the decision to upload an individual medical report is influenced by people’s intention to use the EHR. However, the stigma potential of the diseases mentioned in the reports also influenced this decision. Specifically, uploading diseases with high stigma was associated with higher privacy risk than diseases with low stigma (see Figure 3A). Consequently, stigma potential had a negative influence on the decision to upload records (see Figure 4A), despite generally high intentions to use the EHR.

Thus, intention to use predicts the use of the EHR in part, whereas disease-specific factors such as related stigma can override the general intention. This is particularly evident in Figure 4C where the participants who uploaded reports both with high and low stigma had mostly high intentions to use the EHR (scores>4). However, such a clear distribution of intention to use (scores<4) did not emerge in the case of rejection of uploading. Rather, it is notable that the rejected findings are mainly those with high stigmatization potential (majority of red dots/triangles in Figure 4C). This shows that the effect strength of the stigmatization potential (OR 0.154) is significantly greater than that of the intention to use (OR 2.628). The fact that uploading is rejected due to disease-specific stigma despite high intention to use supports the assumptions of an intention-behavior gap in EHR use [18].

The time course of the diseases had no influence on the decision to upload an individual record. Findings with chronic and acute diseases were uploaded by the majority of participants and with approximately equal frequency (see Figure 3B).

For both use cases, case 1 (patients with multimorbidity) and case 2 (patients after first contact), the results suggest that disease-specific stigma seems to exert an inhibiting influence on the decision to upload. In contrast, the time course only played a role in use case 1, where people interact with multiple reports at a time [31], but not when they interact with only one medical report (use case 2). This difference may be explained by the fact that patients’ “health concerns” have a positive influence on their intention to share health data with others [22]. When faced with multiple medical reports, patients may be more aware and concerned about interactions between chronic diseases, because they more strongly affect the patient’s health both now and in the future; consequently, the willingness to upload reports about chronic diseases increases. With a single report, interactions between diseases are less present, which means that the time course of a disease may play a reduced role in the decision to upload a record.

### Implications

Both the intention to use and the stigma potential of diseases seem to influence whether patients upload an individual medical report to the EHR. Thus, in addition to increasing people’s general intention to use the EHR via marketing and information, transparent and easy-to-comprehend information about the safety standards of the EHR (eg, for encrypting data) and the protection of medical records (eg, the control of access rights) are warranted, even for populations that are already in favor of using the EHR. Such combined interventions may help to reduce security concerns and enable realistic risk assessments of a data leak to ultimately ensure reliable use of the EHR as a key technology in any digitalized health care system.

### Limitations and Future Directions

We deliberately excluded participants who already had a medical history with the diseases addressed in the stimuli to avoid bias in their responses. Individuals living with a stigmatized disease are more cautious to disclose the information, especially if the disease is not immediately apparent [32,43]. The question arises to what extent the behavior of stigmatized individuals can be simulated under experimental conditions, provided that participants do not exhibit stigmatized characteristics. To further strengthen the validity and generalizability of our results, a follow-up study should examine the perspective of already affected individuals and compare the findings with the results of this study.

Another limitation is that the chronic and acute disease patterns used in the stimuli are not readily comparable. We decided to use the diseases listed in Table 1 as stimuli because they achieved the expected effects in the preliminary study [31]. We could only partially replicate these findings in the present study. For future studies, it would make sense to use diseases that can be more readily compared in terms of their stigma potential and time course (eg, gonorrhea and HIV or a wrist fracture and arthritis) to further strengthen the generalizability of the present findings.

Another limitation is that the distribution of our sample in terms of gender, age, and level of education does not correspond to that of the average German population. In particular, the level of education of our sample was above average. Although we were unable to detect any effects of the control variables age, gender, and level of education in the analysis, the results of this study should be validated with a more representative sample in the future.

### Conclusions

In our study, we investigated which disease-specific factors influence whether medical reports are uploaded to the EHR in a German setting. To answer this question, we varied the stigma potential and the time course of diseases in medical reports and controlled for the influence of participants’ intention to use the EHR on uploading behavior. We demonstrated that intention to use had a positive effect on the decision to upload a report. In addition, we found that the stigma potential of the disease listed on the medical reports can inhibit uploading behavior. In particular, we found that the intention to use the EHR may be offset by the stigma potential of a specific record.

In summary, despite the fact that 3 out of 4 Germans state that they intend to use the EHR [11], actual use of this technology may depend on disease-specific factors. Consequently, to ensure successful implementation of the EHR, stakeholders in the health system should not only promote the EHR per se but further develop formats and evaluate them with the help of user testing that provide transparent and easy-to-comprehend information about the standards of data security and control in the EHR. Only in this way can users realistically assess the risks associated with individual EHR use and make an informed decision for (or against) EHR use.

## Appendix

Multimedia Appendix 1 Medical findings used as stimuli.

Multimedia Appendix 2 Questionnaire.

Multimedia Appendix 3 Disease descriptions.

Multimedia Appendix 4 CONSORT-EHEALTH checklist (V 1.6.1).

## Abbreviations

CONSORT: Consolidated Standards of Reporting Trails
EHR: electronic health record
OR: odds ratio
TAM: technology acceptance model
TU: Technische Universität
UTAUT: unified theory of acceptance and use of technology
