# Supplementary material for: Influence of Disease-Related Stigma on Patients’ Decisions to Upload Medical Reports to the German Electronic Health Record: Randomized Controlled Trial
Source: JMIR Hum Factors. 2024 Apr 10;11:e52625. doi: 10.2196/52625 (PMC11043923; doi:10.2196/52625)
Supplement: Multimedia Appendix 1 [file humanfactors_v11i1e52625_app1.pdf]

Alexianer
ST. HEDWIG-KRANKENHAUS

MVZ St. Hedwig-Krankenhaus Berlin
Psychiatrie und Psychotherapie

Große Hamburger Str. 5–11
10115 Berlin

Akademisches Lehrkrankenhaus der
Charité- Universitätsmedizin Berlin

Prof. Dr. med. Dr. phil. Julia
Wegmann - Klinikdirektorin

Tel. (030) 450 537 002
Fax (030) 450 537 921

St. Hedwig-Krankenhaus · DBBZ · Große Hamburger Straße 5-11 · 10115 Berlin

Frau
Dr. med. Annika Kaspar
Fachärztin für Allgemeinmedizin
Schillerstraße 23
10625 Berlin

Befundbericht Berlin, den 28.06.2022

Betrifft: Mustermann, Max

Sehr geehrte Frau Kollegin, sehr geehrter Herr Kollege,

wir berichten über den Patienten Max Mustermann, der sich in unserer stationären Behandlung befand.

Diagnose: Mittelschwere bis schwere depressive Episode (ICD-10: F32. 1-2)

Diagnostik: Gepflegtes Erscheinungsbild. Kleidungsstil altersgemäß. Im Kontaktverhalten mitteilungsbereit und angepasst. Sprachmodulation unauffällig. Stimme leise. Wach und bewusstseinsklar. Zu allen Qualitäten vollständig orientiert. Lang- und Kurzzeitgedächtnis subjektiv und objektiv unauffällig. 3 von 3 Begriffen werden nach 10 Minuten erinnert. Subjektiv schwere, objektiv leichte Konzentrationsstörungen. Auffassung ungestört. Gute Abstraktion in der Sprichwortprüfung. Im formalen Denken geordnet mit unauffälliger Denkgeschwindigkeit. Keine Sinnestäuschungen. Keine inhaltlichen Denkstörungen. Meinhaftigkeit des Denkens erhalten. teilweise Derealisationserleben. Zukunftsängste sowie Sorgen bez. der körperlichen Gesundheit. Keine Zwänge. Stimmung subjektiv gedrückt, Gefühl innerer Leere. Stimmung objektiv schwer gedrückt, verzweifelt und hoffnungslos. Deutliche Insuffizienzgefühle. Schwer reduzierte affektive Schwingungsfähigkeit. Antrieb mittelschwer reduziert, kompletter Verlust von Interesse. Vermindertes Vitalgefühl. Psychomotorisch unauffällig. Vereinzelt Suizidgedanken, im Moment von akuter Suizidalität distanziert. Krankheitseinsicht und Behandlungsbereitschaft gegeben.

Procedere: Eine therapeutische und psychopharmakologische Behandlung ist indiziert. Dem Patienten wird ein stationärer Aufenthalt in einer psychiatrischen Fachklinik angeraten.

Mit freundlichen Grüßen,

Dr. med. Joachim Hagedorn

Bundeswehrkrankenhaus Berlin
Akademisches Lehrkrankenhaus der Charité

Sanitätsdienst
Wir. Dienen. Deutschland.

Scharnhorststraße 13
10115 Berlin

Telefon 030 2941 2289
AllgFspNrBw (90) 8402 2289

berlin.bwkrankenhaus.de

Klinikum III Dermatologie / Venerologie

Chefarzt
Dr. med. Jörg Ziszlak, OTArzt

Sekretariat 030 2941-1301
Telefax 030 2941-1340

Terminvereinbarung 030 2941-1333
Privatsprechstunde 030 2941-1331

BwKrhs Berlin, Dermatologie, Scharnhorststraße 13, 10115 Berlin

Dr. med. Annika Kaspar
Fachärztin für Allgemeinmedizin
Schillerstraße 23
10625 Berlin

Befundbericht

Berlin, den 25.03.2022

Sehr geehrte Kolleginnen und Kollegen, in der Folge berichten wir über unseren gemeinsamen Patienten Max Mustermann, der sich am 25.03.2022 in unserer ambulanten Behandlung befand.

Diagnose: Gonokokkeninfektion, ICD-10: A54.6

Anamnese: die Vorstellung des Patienten erfolgte wegen Schmerzen an den Genitalien und Ausfluss. Die Beschwerden bestünden seit zwei Tagen, zwei Tage zuvor hatte der Patient ungeschützten rezeptiven Geschlechtsverkehr mit einem ihm unbekannten Partner. Bisher hautgesund, bisher zudem keine gleichartigen Ereignisse. Ein HIV–Test sei letztmalig vor zwei Jahren erfolgt.

Befund: bei der Inspektion in Steinschnittlage sehen wir perianal eine deutliche entzündliche Rötung, zudem Schmerzen bei genitaler digitaler Palpation. Inspektorisch reicht das entzündliche Erythem über die Linea dentata bis circa 3 cm ab ano. Zudem begleitende Kryptitis mit gelblich schmierigen Belägen. Die Palpation der Leistenlymphknoten zeigt beidseitig vergrößerte Lymphknoten mit Druckschmerzhaftigkeit. Das übrige Genitale sowie die Dammregion zeigen sich inspektorisch unauffällig.

Diagnostik: eine Blutentnahme zur Bestimmung von HIV, Hepatitis B & C sowie ein Suchtest auf Syphilis sind erfolgt. Es erfolgte die Entnahme eines Abstriches aus dem Sekret, hiernach Verbringen auf einen Objektträger. Lichtmikroskopisch zeigt sich in der Gram–Färbung das Vorhandensein von zahlreichen Leukozyten und Deckzellen, teils mit typischen, gramnegativen intrazellulär lokalisierten Diplokokken als Beweis einer Gonorrhoe.

Procedere: mit dem Patienten wurde der Befund ausführlich erörtert. Aufgrund des Nachweises einer Gonorrhoe erfolgten (es bestehen keine bekannten Allergien gegen

Antibiotika) die intravenöse Gabe von 2 g Ceftriaxon sowie 1,5 g Azithromycin per os. Eine Partnermitbehandlung, so dieser kontaktiert werden kann, wurde dringend angeraten. Der Patient wurde zur Kontrolle des Befundes und zur Besprechung der ausstehenden Untersuchungen aus dem Blut in sieben Tagen wieder einbestellt.

Wir verbleiben mit freundlichen kollegialen Grüßen,

Dr. med. Jörg Ziszlak

IFLb

IFLb LABORATORIUMSMEDIZIN BERLIN GMBH

Dr. med. Peter Flath

Windscheidstraße 18
10627 Berlin

Telefon: +49 30 327 903 158
Telefax: +49 30 327 903 100

www.iflb.de info@iflb.de

IFLb Laboratoriumsmedizin Berlin GmbH
Dr. med. Hannelore Lürscheidt
Windscheidstraße 18, 10627 Berlin

Dr. med. Annika Kaspar
Fachärztin für Allgemeinmedizin
Schillerstraße 23
10625 Berlin

Befundbericht
vom: 01.02.2023

Endbefund

Patient: Max Mustermann
Geschl.: männlich
Patient-ID: 25ON44786952

Abnahme: keine Angabe
Eingang: 01.02.2023 12:23
Ausgang: 01.02.2023 17:59

Diagnose: Die Werte der GOÄ 3560 und 3561 liegen deutlich über der Norm und weisen eine Erkrankung an primär insulinabhängiger Diabetes mellitus (Typ-1-Diabetes) nach.

ICD-10 Klassifikation: E10

GOÄ	Untersuchung	Ergebnis		Mat.	Einheit	Referenz (m)	Grafik
	großes Blutbild						
3550	kleines Blutbild						
	Leukozyten	4.6		ED	/nl	4.0 – 10.0	
	Erythrozyten	5.3		ED	/pl	4.4 – 5.9	
	Hämoglobin	15.4		ED	g/dl	13.5 – 17.5	
	Hämatokrit	43		ED	%	40 – 53	
	MCV	**81**	-	ED	fl	82 – 98	
	MCH	29		ED	pg	27 – 33	
	MCHC	36		ED	g/dl	31 – 36	
	RDW (Ery-Verteilungsbreite)	13.5		ED	%	11.6 – 16.0	
	Thrombozyten	**130**	-	ED	nl	140 – 400	
3551	apparatives Diff.						
	Neutrophile	44		ED	%	40 – 75	
	Lymphozyten	45		ED	%	20 – 45	
	Monozyten	8		ED	%	bis 14	
	Eosinophile	3		ED	%	bis 7	
	Basophile	0		ED	%	0 – 1	
3520	Eisen	125		SE	µg/dl	53 – 167	
3575	Transferrin	247		SE	mg/dl	200 – 360	
	TFS Transferrinsättigung	36			%	16 – 45	
3560	Blutzucker nüchtern	**193**	+	SE	mg/dl	55 – 110	
3561	HbA1c	**8.2**	+	ED	%	4.4 – 6.1	
	HbA1c (IFCC)	**66**	+	ED	mmol/mol	20 – 42	
	mittlerer Blutzucker (ADAG) ermittelt aus HbA1c	189		ED	mg/dl		

GOÄ	Untersuchung	Ergebnis		Mat.	Einheit	Referenz (m)	Grafik
3562H1	Cholesterin	149		SE	mg/dl	bis 200	
3563H1	HDL-Cholesterin	**36**	-	SE	mg/dl	> 40	
	Cholesterin-HDL-Quotient	4.2				bis 5.0	
3584H1	LDL-Cholesterin	87		SE	mg/dl	< 150	
	LDL-HDL-Quotient	2.5				bis 4.0	
		< 2.5 niedriges Risiko<br>2.5 – 4.0 intermediales Risiko<br>> 4.0 hohes Risiko					
3565H1	Triglyceride	137		SE	mg/dl	< 150	
3585H1	Kreatinin	0.9		SE	mg/dl	bis 1.3	
	GFR (MDRD-Formel)	> 90		SE	ml/min	> 63	
		glomeruläre Filtrationsrate (GFR) nach der MDRD-Formel für Erwachsene weißer Hautfarbe					
3584H1	Harnstoff	28		SE	mg/dl	< 50	
3583H1	Harnsäure	5.6		SE	mg/dl	bis 7.0	
3573H1	Gesamteiweiß	7.2		SE	g/dl	6.4 – 8.3	
3581H1	Bilirubin gesamt	0.9		SE	mg/dl	< 1.1	
3582	Bilirubin direkt	0.2		SE	mg/dl	bis 0.3	
	Bilirubin indirekt	0.7		SE	mg/dl		
3594H1	GOT (AST)	**52**	+	SE	U/l	< 50	
3595H1	GPT (ALT)	**83**	+	SE	U/l	< 50	
3592H1	GGT	**84**	+	SE	U/l	bis 60	
3587H1	AP alkalische Phosphatase	81		SE	U/l	< 130	
3589H1	CHE Cholinesterase	9171		SE	U/l	4620 – 11500	
3597H1	LDH-Laktatdehydrogenase	247		SE	U/l	bis 250	
3590H1	CK Creatinkinase	110		SE	U/l	bis 190	
3591H1	CK-MB	11		SE	U/l	< 25	
3588H1	Amylase	57		SE	U/l	< 110	
3598H1	Lipase	51		SE	U/l	< 65	
3558	Natrium	136		SE	mmol/l	135 – 148	
3557	Kalium	**5.7**	+	SE	mmol/l	3.5 – 5.5	
3555	Calcium	2.2		SE	mmol/l	2.1 – 2.6	
3580H1	Phosphat	0.93		SE	mmol/l	0.84 – 1.45	
3621	Magnesium	0.82		SE	mmol/l	0.70 – 1.05	
3572	IgE	**153**	+	SE	U/ml	bis 25	
						Graubereich bis 100	

Ziffern — ID-Nr. 25ON44786952 vom 01.02.23 — Punkte im Auftrag 0 — Punkte im Quartal 0

		3597H1								
3550	3585H1	3590H1	GRBB	X		AppDiff	X	CkMb	11	
	3584H1	3591H1	KLBB	X		'Neut	44	Na	136	
3551	3583H1	3588H1	Leuko	4.6		'Lymph	45	K	**5.7**	+
3620	3573H1	3598H1	Ery	5.3		'Mono	8	Ca	2.2	
3575	3581H1	3558	Hb	15.4		'Eos	3	P	0.93	
3580	3582	3557	Hkt	43		'Beso	0	L-Mg	0.82	
3581	3594H1	3555	Mcv	**81**	-	FE	125	Bz1	**193**	+
3562H1	3595H1	3580H1	Mch	29		Krea	0.9	Hba1c	**8.2**	+
3563H1	3592H1	3621	Mchc	36		Hst	28	4Hba1c	**66**	+
3564H1	3587H1	3572	Rdw	13.5		Hs	5.6	6Ag	189	
3565H1	3580H1		Thromb	**130**	-	Ck	110	Chol	149	

Patient/in

Max Mustermann

Behandelnder Arzt:

Dr. med. Johanna Fichtner

Vivantes
Klinikum Am Urban

Zentrale Notaufnahme/ Rettungsstelle und interdisziplinäre Kurzaufnahmestation – INKA

Dieffenbachstr. 1

10967 Berlin

Tel: (0)30 160 2 295-30, Fax: -32

Behandlung vom: 18.03.2022 11:24

bis: 18.03.2022 12:39

Zuweiser: selbst

Transportmittel: privat

Mobilität: gehfähig

KV-Karte vorgelegt: ja

Entlassungsbericht vom 18.03.2022

Angehörige anwesend: nein

Name: -

Tel: -

Anamnese

Gestern als behelmter Radfahrer mit 20kmh auf beide Hände gestützt. Nun Schmerzen und Schwellung re. HG.

Vormedikation

Klinischer Befund

Re. Arm: DS und Schwellung über dist. Radius. DS und Schwellung über Tabatiere. MHK frei. Radiusköpfchen und Clavicula frei.

Ergebnis der ärztlichen Erstversorgung

durch: Johanniter Unfallhilfe

Aktuell besteht eine Arbeits- bzw. Differentialdiagnose, die eine Krankenhausbehandlung erforderlich macht.

Befunde

Rö-HG in 2 E rechts, Rö-HG nach Stecher: dist. Radiusfraktur rechts mit Gelenkbeteiligung ohne Dislokation

Labor

Vitalparameter

Blutdruck **Herzfrequenz**

RR mm/Hg HF/min arrhythm. GCS Zeit

/ ☐

Pulsoxymetrie: %

☐ mit Sauerstoff ☐ ohne Sauerstoff

Atemfrequenz: /min

Temperatur:

au: °C r: °C

BZ-Stix: mg %

Alkohol (i.d.A.) ‰

U-Stix:

Geriatrisches Screening

ISAR Score

Diagnosen

S52.50G Artikuläre distale Radiusfraktur, geschlossen rechts (A0 23-B1)

Therapie und Verlauf

Röntgen

Anlage dors. UA-Gipsschale

Medikamente Auswahl Gabe um

☐ **Aufnahme im Haus** ☐

☒ **INKA**

Entlassung

☒ nach Hause ☐ Heim

☐ anderes KH ☐ aus INKA

☐ der Patient hat die Behandlung gegen ärztlichen Rat abgebrochen

<table>
<tr><td>Empfehlungen
Kühlung und Schonung
Ibuprofen 600 mg 1-1-1m Pantozol 40mg 1-0-0, für einige Tage fest dann nach Bedarf WB in der Sprechstunde am 21.03.2022 um 09:00 Uhr.</td><td rowspan="3">Hausarzt

Dr. med. Annika Kaspar
Fachärztin für Allgemeinmedizin
Schillerstraße 23
10625 Berlin

(0)30 397 48850</td></tr>
<tr><td>Wiedervorstellung bei Verschlechterung
Die Widervorstellung bei Verschlimmerung oder ausbleibender Besserung bei Ihren weiterbehandelnden Ärzten, beim ärztlichen Bereitschaftsdienst (Tel.:116 117) oder in der Rettungsstelle ist dringend empfohlen!

In einer akut lebensbedrohlichen Situation rufen Sie bitte unter der Nummer 112 den Rettungsdienst der Berliner Feuerwehr an.</td></tr>
<tr><td>Datum / Unterschrift
18.03.2022</td></tr>
</table>
