# Supplementary material for: Influence of Disease-Related Stigma on Patients’ Decisions to Upload Medical Reports to the German Electronic Health Record: Randomized Controlled Trial
Source: JMIR Hum Factors. 2024 Apr 10;11:e52625. doi: 10.2196/52625 (PMC11043923; doi:10.2196/52625)
Supplement: Multimedia Appendix 2 [file humanfactors_v11i1e52625_app2.docx]

**Questionnaire**

**Manipulation check** **(perceived risk):** Rate the following statement: If the information in this finding fell into the wrong hands, it would do me a lot of damage.

- Source: von Kalckreuth et al., 2023 [31]

**Manipulation check** **(perceived benefit):** Rate the following statement: I think it is very beneficial that physicians who treat me have access to this finding through the electronic health record (EHR).

- Source: von Kalckreuth et al., 2023 [31]

**Intention to use**

**IU01**: I should use the EHR as soon as possible.

**IU02**: I would use the EHR.

**IU04**: I would not hesitate to use the EHR.

- Source: von Kalckreuth & Feufel, 2023 [23]

**Behavioral decision**: Would you like to load the diagnosis just shown into your electronic health record?

- Source: von Kalckreuth et al., 2023 [31]

**Demographics**

**Age**: Please enter your age.

**Gender (m/f/d):** Please indicate your gender.

**Education**: Please enter your highest qualification.

- No degree
- School leaving certificate
- Secondary school certificate
- General qualification for university entrance
- Vocational training
- University degree (bachelor’s or master’s)
- other

**Experience with mHealth apps**: How often do you use the electronic health record?

- Never heard of the EHR.
- Heard of the ePA once, but never used it.
- Tried it once.
- I use it regularly.

**Reability Check:** Is there any reason why we should NOT use your data? You will be paid regardless of your response.

- Yes, I rushed through.
- Yes, I did not really read the questions.
- Yes, I choose random answers.
- Yes, for other reasons.
- No, you can use my data.
